# Application of Machine Learning for FOS/TAC Soft Sensing in Bio-Electrochemical Anaerobic Digestion

**DOI:** 10.3390/molecules30051092

**Published:** 2025-02-27

**Authors:** Harvey Rutland, Jiseon You, Haixia Liu, Kyle Bowman

**Affiliations:** 1 School of Computer Science, Electrical and Electronic Engineering, and Engineering Maths, University of Bristol, Bristol BS8 1QU, UK; 2 Bristol Robotics Laboratory, University of the West of England, Bristol BS16 1QY, UK; jiseon.you@uwe.ac.uk; 3 School of Computing and Creative Technologies, University of the West of England, Bristol BS16 1QY, UK; haixia.liu@uwe.ac.uk; 4 School of Life Sciences, University of the Westminster, London W1W 6UW, UK; k.bowman1@westminster.co.uk

**Keywords:** machine learning, deep learning, microbial electrolysis cell anaerobic digestion, FOS/TAC

## Abstract

This study explores the application of various machine learning (ML) models for the real-time prediction of the FOS/TAC ratio in microbial electrolysis cell anaerobic digestion (MEC-AD) systems using data collected during a 160-day trial treating brewery wastewater. This study investigated models including decision trees, XGBoost, support vector regression, a variant of support vector machine (SVM), and artificial neural networks (ANNs) for their effectiveness in the soft sensing of system stability. The ANNs demonstrated superior performance, achieving an explained variance of 0.77, and were further evaluated through an out-of-fold ensemble approach to assess the selected model’s performance across the complete dataset. This work underscores the critical role of ML in enhancing the operational efficiency and stability of bio-electrochemical systems (BES), contributing significantly to cost-effective environmental management. The findings suggest that ML not only aids in maintaining the health of microbial communities, which is essential for biogas production, but also helps to reduce the risks associated with system instability.

## 1. Introduction

Anaerobic digestion (AD) is an effective biotechnology for converting a variety of organic wastes into biogas. However, the stability and efficiency of AD processes are challenged by factors such as substrate variability, organic loading rates, and the accumulation of substances like volatile fatty acids (VFAs), which can lead to inhibition, causing fluctuations in methane production and pH [1].

MEC-AD systems have been shown to achieve higher methane yields compared to standard AD practices. Systems integrating low-voltage (poised under 2 V) electrodes within the reactors have demonstrated greater operational stability under lower pH conditions, which is beneficial for maintaining the health and efficiency of the microbial communities responsible for biogas production [2,3,4]. Furthermore, the integration of MECs with AD systems enhances substrate degradation and increases biogas production. MEC-AD systems additionally offer potential improvements in process control by enabling real-time monitoring, which correlates electrical signals with substrate concentrations, significantly enhancing operational efficiency [5].

VFAs are critical substrates in the AD process, with the most common form of methanogenesis in conventional AD being acetotrophic methanogenesis, where acetate is the sole carbon source. VFA concentration serves as a key indicator of process health and stability. Monitoring VFAs is essential for preventing the digester from experiencing process imbalances that could lead to system failures, such as acidosis. Accurate and timely measurements of VFA concentrations help operators maintain optimal operating conditions, thus maximising biogas production and improving resource efficiency. High VFA levels can inhibit methanogens, thereby obstructing methane synthesis.

The FOS/TAC ratio, where FOS (Flüchtige Organische Säuren) represents the concentration of VFAs and TAC (Totales Anorganisches Carbonat), characterises the system’s total alkalinity or buffering capacity and is a crucial metric for maintaining equilibrium within AD processes. Ideally, this ratio should fall between 0.3 and 0.4, although the optimal range may differ from one system and substrate to another [6,7]. Monitoring deviations in the FOS/TAC ratio provides early warning signs of process imbalances before substantial pH changes occur. For instance, an elevated FOS/TAC ratio may indicate excessive VFAs or inadequate alkalinity, conditions that can lower pH and hinder the activity of methanogenic bacteria. Conversely, a lower ratio may signal insufficient organic loading and thus diminished biogas production. By closely observing this ratio and making necessary adjustments such as modifying feed rates, introducing buffering agents, or altering other process parameters, operators can maintain optimal conditions, ensure efficient methane generation, and prevent acidification-related disruptions.

Despite its significance, continuous monitoring of the AD process, including FOS/TAC measurements, poses economic and logistical challenges. The use of live sensors for wastewater analysis often requires substantial upfront investment and ongoing expenses for maintenance and calibration [8]. Additionally, on-site operators must frequently collect and analyse samples or rely on external laboratories, which not only increases labour but also delays the availability of results [9]. These limitations underscore the potential of soft sensor models, which leverage auxiliary variables to provide real-time optimisation and control. By reducing reliance on costly, time-consuming monitoring methods, this offers lower operational overheads and supports more efficient and responsive management of the AD process [10].

The incorporation of ML approaches into anaerobic digestion is on the rise, primarily for system modelling and to refine the understanding of operational variables like predicting gas yields. These ML approaches span various types, from conventional techniques to deep learning and hybrid models, all demonstrating success in forecasting methane production under different conditions. Anaerobic digestion is characterised by significant dimensionality and intricacy, necessitating the monitoring of numerous parameters crucial for operational guidance. By encapsulating the complex interrelations of biological and chemical processes, ML models enable the prediction of parameters that would traditionally depend on in situ sampling and labour-intensive analyses, where operators must visit sites to collect samples for subsequent evaluation. A reactor that is inadequately monitored is susceptible to instability due to VFA accumulation if the feedstock and operation conditions are variable, for example due to seasonal changes. In practice, this may necessitate taking the reactor offline for recovery, during which time waste remains untreated and gas production is halted.

Recent advances in soft-sensing technologies have significantly enhanced the monitoring and optimisation of AD processes. By enabling the real-time prediction of key parameters such as VFAs, chemical oxygen demand (COD), and biogas yield, these methods address many of the challenges posed by traditional monitoring approaches [11]. In previous work, genetic programming and ANNs were trained on synthetic data to emulate real-world conditions, thereby improving both the precision and reliability of VFA monitoring [12]. Another promising avenue involves reverse modelling with the ADM1 model to estimate substrate characteristics from digester output data, thereby enabling more effective input management and greater process stability [13]. Other dynamic soft sensors have been developed to use spatiotemporal graph convolutional networks (CNN) that draw on both spatial and temporal data to improve VFA concentration predictions and better accommodate industrial process variability [14]. Altogether, these methodologies underscore the indispensable role of advanced data analytics in boosting the efficiency and sustainability of AD systems.

This study integrates soft sensing and MEC technologies into AD processes. It employs ML to predict FOS/TAC using real-time data from a pilot MEC-AD system. By leveraging the rapid stabilisation inherent to MEC-AD systems, this approach reduces the length of training datasets, accelerates initial operations, and enhances the viability of pilot studies. This strategy involves the development and assessment of predictive models prior to integrating live FOS/TAC parameter predictions into operational workflows, offering an effective, low-cost, in situ option for monitoring system stability. In contrast to current investigations with AD systems which have investigated parameter prediction on extensive, long-term datasets or synthetic simulations, these methods provide immediate feedback for parameter estimation, increasing adaptability while decreasing reliance on human oversight.

In demonstrating the feasibility of soft sensing in MEC-AD systems, this work highlights a data-driven method for reducing operational demands and associated costs. Further investigation may reveal the viability of transferring learning between multiple MEC-AD facilities. The emphasis on predicting FOS/TAC opens the door for broader applications across the wastewater sector. By using digital soft triggers activated by real-time data, this approach not only improves operational efficiency but also encourages more responsive and economical monitoring and control solutions. Integrating this sensing and detection framework into the workflow of AD and MEC-AD operations can unlock new potential for cost-effective, scalable implementation in wastewater treatment applications.

## 2. Results and Discussion

### 2.1. Feature Analysis Evaluation

**Figure 1:**
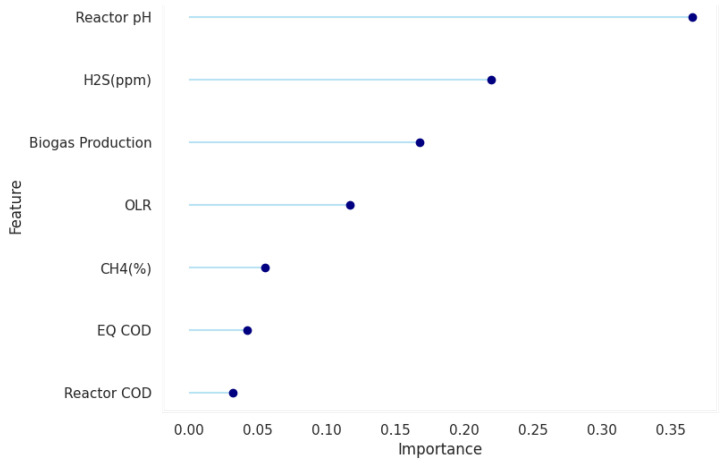
Reduced feature space selected by the random forest feature importance model.

Feature analysis revealed that the importance scores of features ranked below the seventh position decreased only marginally, prompting the selection of the top seven most important features, which predominantly contributed to the overall model performance. The feature analysis was then repeated on this subset of data, and Figure 1 highlights the features implemented for training and optimisation in the pipeline. The target variable and the highlighted features aligned with established knowledge of FOS/TAC prediction, particularly with regard to pH, which emerged as highly important. This aligns with the principle that VFA build-up relative to buffer capacity is reflected in pH values, whereby a low pH inhibits methanogenic activity and consequently reduces methane production—an effect observed in the monitored biogas output. Additionally, H_2_S ranked second in importance, reflecting that in the breakdown of organics, sulphate-reducing bacteria (SRBs) compete with methanogens for substrates such as H_2_ and acetate; when methanogens are inhibited, SRB activity increases, leading to higher hydrogen sulphide production [15,16]. Other reactor-related features, such as the chemical oxygen demand (COD) of the equalisation tank, the COD of the reactor, and the organic loading rate (OLR), conform to operational understanding: fluctuations in COD feed can either inhibit or starve the reactor, causing corresponding spikes or dips in FOS/TAC readings.

### 2.2. Model Comparison

After running the random search on the specified model, it was identified that among various performance metrics, the best results were achieved with the ANN when averaged over the five folds. The result from the best-performing models are listed in Table 1. As 5-fold cross-validation was run independently out of fold, five sets of hyperparameters were generated for each model; the best-performing hyperparameter configurations are listed in Table 2. When comparing the average performance values across all folds, the traditional models achieved a lower explained variance and higher MAE and NRMSE, indicating lower performance.

### 2.3. Fold Investigation

**Figure 2:**
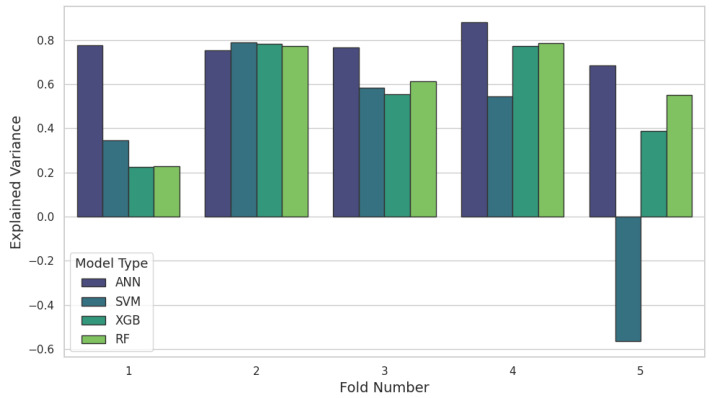
Explained variance for each model evaluated, ordered by test fold.

When looking at a pilot dataset, the data’s temporal nature and the MEC-AD system’s internal characteristics should be considered. Results illustrating the explained variance across the five folds are depicted in Figure 2. The analysis shows that the middle three folds tend to exhibit higher explained variance across all models. Notably, the SVM model underperforms significantly in Fold 5, with an explained variance of −0.56, adversely affecting the average performance, as reported in Table 1. This underperformance in specific folds, particularly Fold 5, can be attributed to sudden shifts in loading conditions and temperature, which pose greater challenges for models lacking the adaptability of ANNs to complex nonlinear dynamics. Traditional models like SVM are especially sensitive to noisy or outlier data, and while models such as random forest and XGBoost display reasonable efficacy during stable periods, their performance declines when operational data deviate from typical conditions.

Analysis of Fold 1 revealed that traditional models suffered significantly from operational inconsistencies during the startup phase. These models likely struggled due to an incomplete representation of startup conditions in the dataset. Noise introduced by initial system adjustments led to reduced accuracy. Another key observation was the negative impact of increased organic loading on the internal temperature of the reactor, which fell below the optimal range for anaerobic digestion operations. This condition was particularly evident in the data from Fold 5, where the models consistently showed poorer performance. This suggests a lack of representation for such adverse conditions in other parts of the training dataset, pointing to a potential gap in the diversity of operational scenarios included during model training. However, this was found to have a lower impact on the performance of the ANNs, which remained stable across all five folds. Techniques such as dropout can aid ANNs avoid overfitting to noisy or outlier data [17]. This enables the models to be more resilient to inconsistent data inputs, allowing ANNs to perform more robustly and reliably in scenarios that inhibit the performance of traditional models, demonstrating their suitability for handling the variabilities of industrial data streams.

Hyperparameter optimisation is important to ensure that the model is capable of capturing the complexities of the training data without overfitting. For the SVM models, a linear kernel with strong regularisation parameters (C=0.01, ϵ=0.01) proved most effective, indicating that a simple decision plane was optimal. In Fold 2, this configuration achieved an MAE of 0.0614, an NRMSE of 0.678, and an explained variance of 0.788. However, applying the same hyperparameters in Fold 1 resulted in a higher MAE (0.2178) and lower explained variance (0.346). This highlights how temporal characteristics and varying data distributions can significantly influence model performance. Assessing results across the folds suggests that the linear kernel with appropriate regularisation offers better robustness against such variations.

Tree-based models like XGBoost and random forest with simpler configurations generally showed better performance. The best-performing XGBoost model in Fold 4 utilised a lower max depth of 2200 estimators, and a reduced learning rate of 0.03, achieving an MAE of 0.0629 and an explained variance of 0.774. Increasing model complexity in other folds did not necessarily improve performance, suggesting that the models might be fitting to specific temporal characteristics evident in those folds rather than generalising well across the dataset. Similarly, the optimal random forest model, also in Fold 4, employed 1300 estimators, a max depth of 10, and a max features parameter of 0.7, introducing a high level of randomisation and cutoff between the trees. This configuration resulted in an MAE of 0.0586 and an explained variance of 0.7843. Despite using similar hyperparameters, performance varied across other folds, emphasising the impact of data variability and the need for tailored hyperparameter tuning.

**Figure 3:**
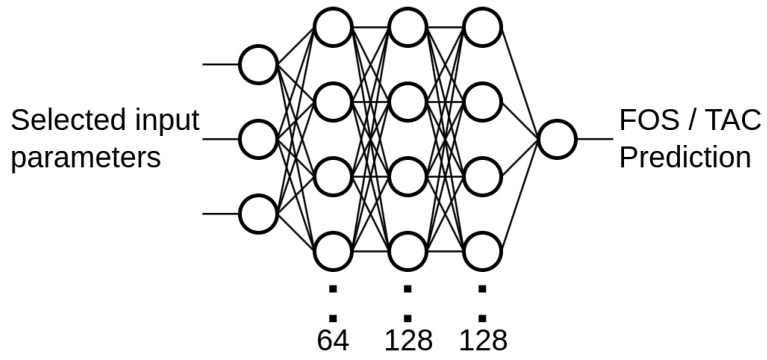
Neural network structure selected for out-of-fold predictions in ensemble evaluation.

ANN models consistently outperformed traditional models across all folds, demonstrating robustness and superior predictive accuracy. The optimal ANN configuration in Fold 4, featuring a three-layer architecture with neuron counts of [64, 128, 128], is highlighted in Figure 3. This configuration was trained with a learning rate of 0.0013. This model achieved an MAE of 0.0428, an NRMSE of 0.3488, and an explained variance of 0.8784. Other folds with varying architectures and learning rates also performed strongly, underscoring the ANN’s ability to capture complex nonlinear relationships inherent in the data. The variation in network depth, neuron counts, and learning rates across folds highlights the importance of carefully configuring these parameters to enhance model generalisation and predictive accuracy.

Overall, these results suggest that models with simpler architectures and appropriate regularisation tend to generalise better across different data segments. In the context of using these models as soft sensors, the training data available prior to implementation may be limited or may require the transfer of historical data from other trials. Utilising simpler models often prevents overfitting to less relevant features of the data, thereby enhancing the model’s generalisation capabilities when applied to new datasets or different operational settings [18]. The ANNs show consistent performance, suggesting they are well-suited to capturing the underlying dynamics present in the training data. This aligns with findings listed in other review papers that compare application domains [10,19].

### 2.4. Out-of-Fold Predictions for Ensemble Evaluation

Due to the ANN model producing the best predictive results among the models investigated, the optimal results were observed in Fold 4. Over the five folds, different sets of hyperparameter configurations were generated to facilitate the initial implementation and narrow down to a single model for in situ production. To achieve this, the best-performing model structure was used to conduct an ensemble evaluation using out-of-fold predictions derived from *k*-fold cross-validation. The same 5-fold cross-validation strategy, without shuffling, was utilised.

This network architecture comprised three hidden layers with 64, 128, and 128 units, respectively, and employed the ReLU (Rectified Linear Unit) activation function. It was optimised using the Adam optimiser with a learning rate of 0.00129. The Adam optimiser was chosen for its efficiency in training, especially suitable for this soft sensing applications due to its proficiency with datasets characterised by inconsistent events, such as sudden spikes or drops in parameters like organic load [20]. This capability makes it an excellent choice for ensuring accurate and reliable model performance in dynamic environments. The ReLU activation function was selected for its computational efficiency, which allows for capturing non-linear relationships without significant computational demands [21]. To mitigate the effects of random weight initialisation and the stochastic nature of training, five independent instances of the model were trained per fold, each with different random seed initialisations.

The predictions from these models were averaged to produce the final prediction for each fold’s validation set, effectively forming an ensemble basis of performance assessment. Deterministic operations were enforced in TensorFlow. The results from this assessment are summarised in Table 3, showing comparative results to the original hyperparameter training pipeline. Fold 5 produced a significantly lower explained variance compared to the other folds. However, when inspecting an average of all folds, the explained variance was 0.62. Excluding Fold 5, this gives a performance of 0.74, indicating that this model structure is capable of explaining a substantial amount of the variability in the data across all folds.

The use of the ensemble method provides a robust and reliable assessment of the model’s performance. Averaging predictions from multiple models per fold mitigates the impact of random initialisation and stochastic variations during training, leading to more stable predictions and a reduction in the variance of performance metrics. Variations in MAE and NRMSE across folds reflect inherent dataset variability and the challenges associated with modelling complex biochemical processes.

**Figure 4:**
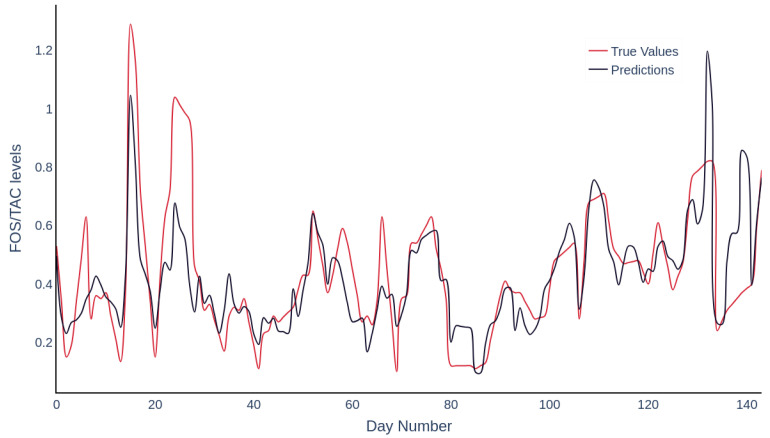
Depiction of the time series analysis comparing actual values to predictions using the selected ANN structure, employing out-of-fold ensemble evaluation. The data are segmented by training folds.

**Figure 5:**
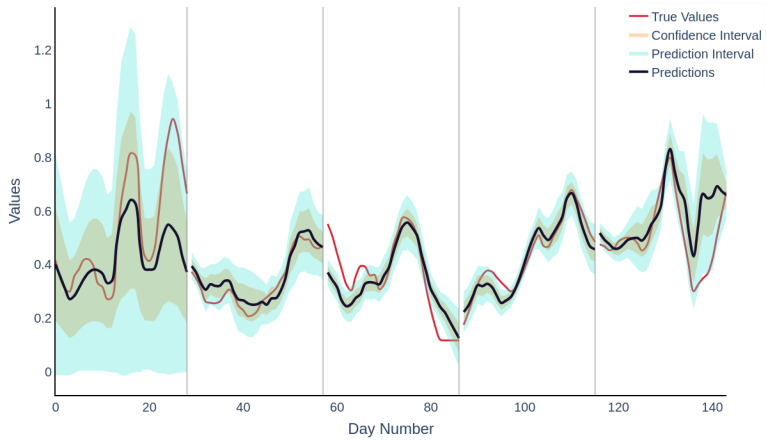
Visualisation presenting a 5-day moving average of the true and predicted data, clearly highlighting variations in confidence and prediction intervals throughout the trial. Additionally, data segmentation by training folds is shown, offering insights into the distribution of data across different training periods.

By preserving the temporal sequence in cross-validation and preventing data leakage, the model is able to learn authentic temporal patterns, which are crucial for deployment in dynamic processing environments. The methodology addresses common challenges in soft sensor development, including limited data availability and risks of overfitting. A depiction of the true versus predicted values, plotted over the course of the trial, is presented in Figure 4. During the initial folding phase, representing the startup phase, undetected events may have compromised data integrity, leading to increased uncertainty in the early predictions of the model. This is evidenced by the expanded confidence intervals and prediction intervals. Figure 5 presents an adjusted plot showing a 5-day moving average along with the prediction and confidence intervals. In the final trial month, reduced offline data sampling frequency necessitated linear interpolation to align online data with offline data, potentially degrading data quality. This is reflected in the broader prediction intervals and diminished confidence levels in later stages, indicative of the model’s decreased predictive reliability due to inconsistent data inputs. Data at both trial ends often showed range extremities, potentially limiting the generalisation capabilities of models trained without these folds.

**Figure 6:**
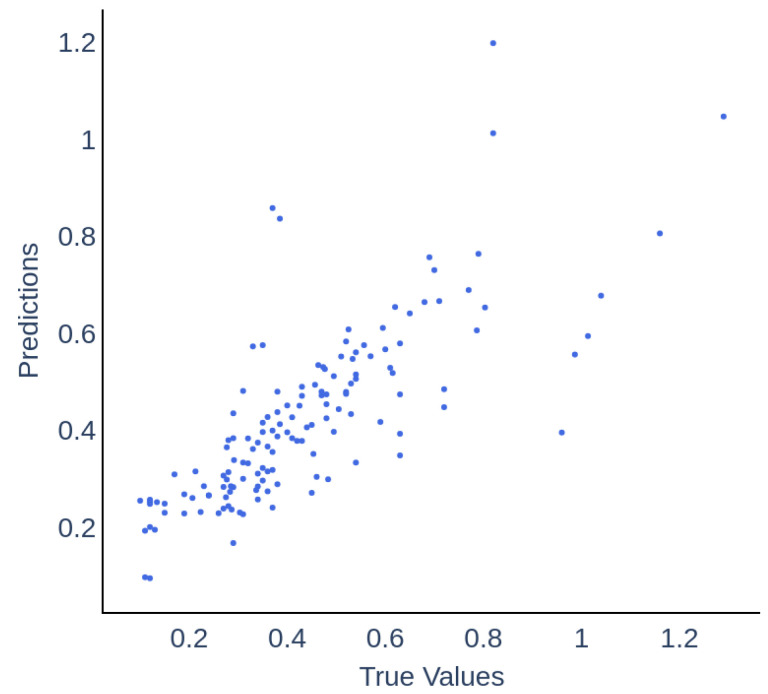
Direct comparison of actual values to predictions from the ANN, including data from all folds in the out-of-fold ensemble evaluation.

A direct comparison between true and predicted values is further compiled in Figure 6. The data points predominantly fall in the range of 0.2 to 0.6 on both axes, corresponding to periods of stable reactor operation during the field trial and indicating a prevalence of lower FOS/TAC ratios in the dataset. There is a noticeable spread in points at higher values, suggesting that the model’s accuracy may diminish as the FOS/TAC ratio increases, a trend that is quantitatively supported by the accuracies and F1 scores listed in Table 4. Specifically, this table illustrates a reduction in model accuracy from 0.79 to 0.58 and in F1 score from 0.89 to 0.73 as the FOS/TAC ratio increases from the 0.3–0.6 range to values greater than 0.6. In this context, accuracy measures the proportion of total predictions that the model correctly identifies, highlighting a decrease in the model’s ability to accurately classify reactor statuses outside the stable operating range. Similarly, the F1 score, which balances precision and recall, shows a decline, suggesting that the model becomes less precise and comprehensive in capturing all relevant instances under higher FOS/TAC conditions. From an operational perspective, even if there is a deviation from exact values at these higher levels, the overall trend can still be discerned. This allows for timely operational interventions based on the general behaviour of the system rather than on precise predictions, helping to maintain system stability and efficiency.

## 3. Materials and Methods

### 3.1. Data Collection

**Figure 7:**
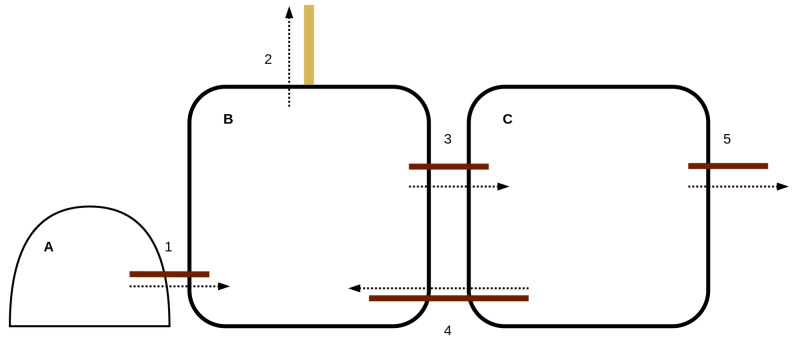
Block-flow diagram depicting the operational reactor setup. Components and flows are labelled as follows: (**A**) waste equalisation tank, (**B**) primary MEC-AD reactor, (**C**) effluent holding tank, (1) feeding inlet, (2) gas outlet, (3) tank 1 outlet, (4) sludge recirculation, (5) effluent outlet.

**Figure 8:**
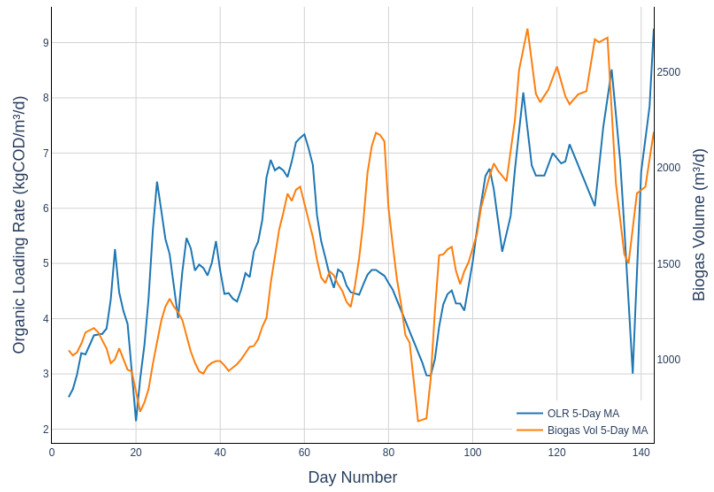
Trends in organic loading rate (OLR) and biogas volume over the trial period. The lines represent 5-day moving averages for both parameters.

This study used data from a five-month pilot trial that employed a miniWASE™ system, provided by WASE from Bristol, United Kingdom. This system is a 4000-litre, four-chamber MEC-AD system. The model focused on the primary chamber of the MEC-AD system, which has a capacity of 1000 litres, and incorporated data from an upstream equalisation tank used to prepare the influent waste. A diagram depicting the reactor configuration, which was used to compile the dataset, is presented as a block flow diagram in Figure 7. The primary MEC-AD reactor was fed from the equalisation tank containing homogenised influent wastewater. Following a feed event, settled solids from the effluent holding tank were recirculated back into the primary MEC-AD reactor. After this, the primary reactor was mixed using gas mixing, recirculating methane from the reactor’s headspace. Throughout the trial, site operators conducted daily monitoring (excluding weekends) for four months, collected digestate samples, and managed operations. The compiled dataset included data from variables tracked by online sensors and lab-based analyses. The parameters listed in Table 5 detail those that can be monitored using online methods, which facilitates the potential use of soft sensors. Table 6 provides a statistical overview of the dataset used in this study, summarising the key monitoring parameters and their variations during the trial period. The average COD of the wastewater was approximately 15,939.9 mg/L, as shown in the mean values, with the maximum observed variation reaching 32,948.0 mg/L, illustrating significant fluctuations in wastewater strength. Throughout the trial, the organic loading rate was progressively increased alongside a reduction in hydraulic retention time, achieving stable operation at 2.3 days. This adjustment is reflected in the biogas volumes shown in Figure 8. The data were adjusted to show a 5-day moving average, providing a clearer depiction of trends over the trial period. During the final month, the frequency of offline data sampling decreased to about every two days during periods of stable operation. To fill in gaps in the dataset, the data were linearly interpolated to synchronise all the online data with the offline parameters.

### 3.2. Investigation Approach

A comparison is presented between traditional and deep-learning-based ML methods. Traditional approaches, including regression analysis and decision trees, often require fewer computational resources, resulting in faster training and prediction times on smaller datasets [22]. However, these approaches can struggle with complex, non-linear relationships and may not scale as effectively for large datasets or adapt as flexibly to new data as deep learning techniques [23].

Deep learning models, notably artificial neural networks (ANNs), excel in handling intricate patterns within large datasets and can automatically extract relevant features, minimising manual feature engineering [24]. They have the capacity to adapt continuously to new data with relatively minor re-engineering efforts. Nevertheless, their lack of transparency can prevent interpretability and validation, especially for high-dimensional data. Ongoing research in explainable artificial intelligence is working to mitigate this challenge and enhance the trustworthiness and usability of deep learning models [25].

The research methodology employed a systematic workflow, beginning with data context analysis, followed by data cleaning, feature analysis, and dimensionality reduction. Subsequent to these initial steps, a variety of modelling techniques were introduced and evaluated, spanning both traditional and deep learning approaches. SVM can effectively filter out noise by ignoring deviations within a certain threshold ϵ. This capability is viable for applications where data can be noisy and relationships between variables are complex or not fully represented in the dataset. SVM balances model complexity with predictive accuracy, which makes it particularly suitable for accurately predicting parameter trends [26].

**Figure 9:**
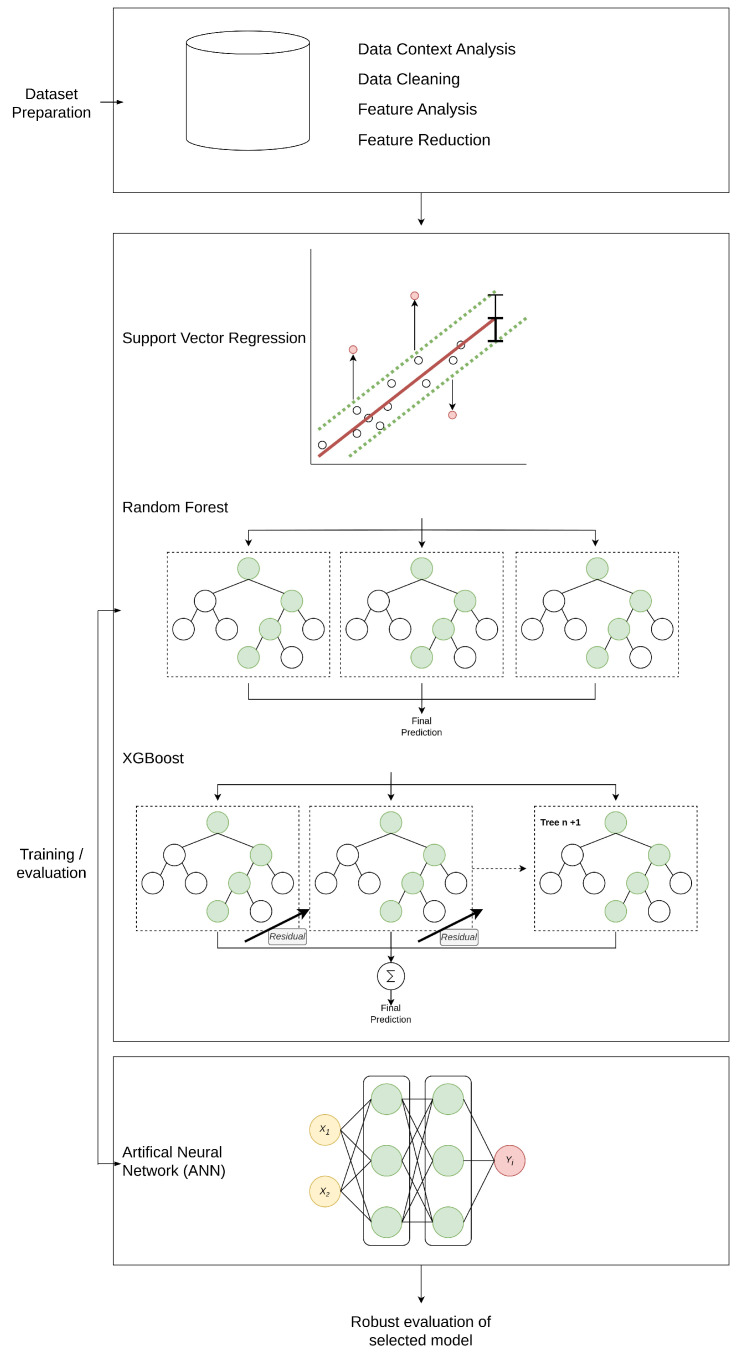
ML investigation processes and model comparisons used in the development of the soft sensing model. Models investigated include SVM, random forest, XGBoost, and ANNs, the best performing model was then selected for more robust analysis.

Random forest (RF) creates an ensemble of decision trees, enhancing generalisation and reducing overfitting by combining multiple estimators [27]. XGBoost extends this approach by applying gradient boosting principles that iteratively refine residual errors from preceding models, incorporating built-in regularisation and flexible hyperparameters [28]. On the deep learning front, ANNs learn data representations through interconnected layers of neurons and adjust parameters via backpropagation to minimise error functions [29]. An overview of this integrated workflow is depicted in Figure 9.

This study focuses on comparing the applicability of these diverse approaches, using data from the MEC-AD system field trial, for in situ FOS/TAC prediction. By examining their training efficiency, predictive accuracy, scalability, and interpretability, this study definitively selects the most appropriate modelling strategy for the given domain.

#### 3.2.1. Evaluation Metrics

To assess the predictive performance, this study employs several standard evaluation metrics. The $R^{2}$ score, or the coefficient of determination, measures the proportion of variance in the dependent variable explained by the model,

$$\begin{array}{cc} R^{2} & =1-\frac{\sum_{i=1}^{n}{\left(y_{i}-{\hat{y}}_{i}\right)}^{2}}{\sum_{i=1}^{n}{\left(y_{i}-\bar{y}\right)}^{2}} \end{array}$$

The normalised root mean square error (NRMSE) provides a scale-independent measure of prediction accuracy,

$$\begin{array}{cc} \mathrm{NRMSE} & =\frac{\sqrt{\frac{1}{n}\sum_{i=1}^{n}{\left(y_{i}-{\hat{y}}_{i}\right)}^{2}}}{\bar{y}-\min\left(y\right)} \end{array}$$

The mean absolute error (MAE) captures the average magnitude of the errors in a set of predictions,

$$\begin{array}{cc} \mathrm{MAE} & =\frac{1}{n}\sum_{i=1}^{n}\left|y_{i}-{\hat{y}}_{i}\right| \end{array}$$

where

$y_{i}$ = actual value of the dependent variable for the *i*-th sample,

${\hat{y}}_{i}$ = predicted value of the dependent variable for the *i*-th sample,

$\bar{y}$ = mean of the actual values,

min(y) = minimum of the actual values,

*n* = total number of samples.

#### 3.2.2. Feature Reduction

Initial manual reduction in the operational dataset was performed to only include data from the equalisation tank and the primary reactor in the MEC-AD system. Further feature importance analysis was conducted using a random forest model, which was selected for its effective handling of complex, interrelated features without the need for transformation or scaling. In the random forest algorithm, feature importance is assessed by calculating the decrease in node impurity across all trees in the forest when a particular feature is used to split the data. The average reduction in impurity provides a measure of the feature’s ability to enhance model accuracy [30,31,32]. The ensemble method used by random forest reduces error variance compared to other methods like linear models or single decision trees, thereby improving the model’s ability to generalise to new data. Unlike linear models that may overlook interactions among features, random forest captures these interactions without requiring explicit specification, making it adept at modelling complex ecological systems. Furthermore, the robustness of random forest against overfitting, even with a large number of features and complex data structures, ensures more reliable and consistent feature importance rankings.

#### 3.2.3. Out-of-Fold Cross Data Training and Evaluation Methodology

To compare the performance of the listed models, an out-of-fold cross-validation approach was employed during training. This ensured a robust evaluation and mitigates potential variability and biases due to data quality or sampling rates from different periods of the field trial dataset. The dataset was partitioned into five folds without shuffling to preserve any inherent structure within the data. In each fold, 20% of the data were reserved for final validation, while the remaining 80% constituted the training set. Nested cross-validation was performed within each training set for hyperparameter tuning. Consequently, each model was validated five times using the optimal hyperparameters selected during the tuning process.

Hyperparameter optimisation for the traditional models was conducted using the RandomizedSearchCV function from the scikit-learn library, version 1.6.1 [33]. This method adopts a stochastic approach by randomly sampling combinations from specified distributions of hyperparameters over a predefined number of iterations. A scoring function was defined to systematically evaluate the performance of each hyperparameter combination. This approach is designed to improve the predictive accuracy of the soft sensor by effectively exploring the parameter space. For tuning the artificial neural network (ANN), the Keras Tuner library, version 1.4.7, was employed due to its effectiveness in systematically exploring the hyperparameter space of neural networks. All software components were implemented using Python 3.

The configuration was set to run up to 60 trials, each with five executions to account for stochastic variability. Within these trials, the number of hidden layers (ranging from 1–4), units per layer (32–128), and learning rate (1 $\times \;10^{-4}$–1 $\times \;10^{-2}$) were varied and evaluated via cross-validation. Averaging across multiple executions further mitigated performance variance. Through this process, we identified the three-layer architecture comprising 64, 128, and 128 units as the most robust model. The Keras Tuner library supports various search strategies, including random search, Hyperband, and Bayesian optimisation, each offering specific advantages in terms of speed and efficiency. In our application, performing multiple executions per trial enhanced the reliability of the results by mitigating variability arising from random initialisation and other stochastic factors.

## 4. Conclusions

This study conducted an empirical investigation using a field trial dataset to approach the challenges and opportunities inherent in implementing data-driven soft sensing models under realistic operational conditions. Contrary to numerous previous studies that depended on extensive datasets derived from prolonged operational periods, the methodology employed demonstrates the feasibility of achieving substantial model performance with a pilot dataset of under 150 days. This feasibility is attributed, in part, to the configuration of the examined process as an MEC-AD system, which can achieve stable operational conditions in a shorter period compared to traditional AD systems. As a result, the requisite lead time for data acquisition prior to deploying a soft sensor is reduced, thus facilitating the integration of these technologies into operation without necessitating prolonged baseline trials. The FOS/TAC levels, observed over three months of stable operation, adapted to changes in organic loading and specific microbial activity. Further investigation should be undertaken to evaluate how models developed under these conditions perform in less stable operational conditions such as those linked to instability. To mitigate these limitations, it is crucial to expand the variety of operational data and integrate datasets from diverse trials. Incorporating advanced data analytics can provide deeper insights into the rarity and uniqueness of the data and the states of the reactor. Additionally, when analysing across multiple datasets, a variety of feature analysis methods should be considered and compared to assess commonalities and differences exhibited by different operational datasets. Methodologies surrounding feature analysis should be expanded to provide further insights into inherent feature importance variability. This expanded analysis will enhance understanding of which features consistently influence model performance and how they vary across different operational conditions, thus supporting the development of more robust and adaptive models. Identifying periods of data scarcity or states of the reactor that are poorly characterised enables a more precise quantification of the model’s uncertainty. This approach aligns with the principles of explainable artificial intelligence (XAI), which emphasises not only transparency but also the reliability of models under varying conditions. When deploying these models in industrial process controls, acknowledging and communicating these uncertainties can ensure that decisions are made with appropriate caution [34].

To further enhance the predictive capabilities of these models, future work could explore the monitoring of microbial communities as a key variable in the ML pipeline for methane yield. Approaches may look to utilise data on microbial shifts within the model pipeline to enhance predictive outputs over extended time periods. By integrating microbial community data, we may facilitate a more comprehensive understanding of bioprocesses, ultimately leading to optimised operational strategies [35,36].

The impact of sampling rate should also be noted, as extended periods of interpolation between data points can negatively affect data quality. Either new methods of gap filling should be investigated or these periods should be removed from the training set. Although the ANN exhibited superior performance in this analysis, the introduction of novel data from additional trials could potentially render other modelling approaches more viable. An increase in data volume could enhance the efficacy of traditional models, thereby increasing their feasibility, particularly in scenarios requiring regular retraining. Furthermore, in contexts where multiple sites operate in real time with frequent model updates, models characterised by lower computational demands may prove more advantageous, especially when implemented across a distributed network. Prolonged intervals of data interpolation may adversely affect model accuracy, underscoring the necessity to either refine gap-filling methodologies or exclude inferior data segments from the training dataset. While ANN demonstrated superior outcomes with the current dataset, the acquisition of supplementary data from other trials might enable traditional models to compete more effectively, potentially rendering them more economically viable and computationally efficient options, representing an imperative consideration for distributed, real-time process monitoring across multiple sites.

Evaluating the robustness and practical advantages of these soft sensors through live trials in a 4000 L scale-up MEC-AD trial system presents a unique research opportunity to advance this technology. With the current developments in ML predictions, these models can now be integrated directly into the operational loop and tested across various operational pilots on the same system. This allows for a continuous assessment of how to integrate these predictive modelling methods into ongoing operations effectively. Highlighting model explainability, continuous retraining, and integration into process controls will ensure that operators can trust and efficiently utilise these predictive tools. Ultimately, the innovation demonstrated in this work leveraging a small dataset facilitated by the rapid stabilisation of an MEC-AD system opens promising pathways for deploying soft sensing solutions in settings where extended data collection periods would be impractical. This stands to accelerate the practical adoption of data-driven analytics in a broader range of real-world operations, targeting implementation in a period close to that of commissioning.

## Figures and Tables

**Table 1:** Average results for all models investigated in out-of-fold five-fold cross-validation, as identified through random search.

Model Type	MAE	NRMSE	Explained Variance
Random Forest	0.12	0.77	0.59
SVM	0.14	0.94	0.34
XGBoost	0.13	0.81	0.55
**ANN**	**0.072**	**0.48**	**0.77**

**Table 2:** Optimal hyperparameter configurations for each model derived from the independent 5-fold cross-validation runs.

Model Type	Best Fold	Corresponding Best Fold Hyperparameters
SVM	2	tol = 0.0001, shrinking = True, kernel = linear, gamma = 0.01, epsilon = 0.01, degree = 2, coef0 = 2.5, C = 0.01
XGB	4	subsample = 0.6, lambda = 2, alpha = 0.1, estimators = 200, maximum depth = 2, learning rate = 0.03, colsample bytree = 1.0
RF	4	estimators = 1300, minimum sample split = 5, minimum samples leaf = 1, maximum features = 0.7, maximum depth = 10
ANN	4	layers = 3, neurons per layer = [64, 128, 128], dropout = none, learning rate = 0.0013, activation = ’relu’

**Table 3:** Model performance metrics across folds.

Fold	MAE	NRMSE	Explained Variance
Fold_1	0.17	0.68	0.65
Fold_2	0.045	0.49	0.78
Fold_3	0.085	0.58	0.67
Fold_4	0.048	0.38	0.86
Fold_5	0.11	1.0	0.15
Average	0.092	0.63	0.62

**Table 4:** Accuracy and F1 Score by range.

Range	Accuracy	F1 Score
0–0.3	0.78	0.87
0.3–0.6	0.79	0.89
>0.6	0.58	0.73

**Table 5:** Monitoring parameters and their units.

Parameter	Unit
Equalisation Tank pH	-
Equalisation Tank COD	mg/L
Feed Volume	L
Organic Loading Rate	kg COD/$m^{3}\cdot$ day
Reactor pH	-
Reactor COD	mg/L
Current	A
Temperature	°C
CH_4_	%
H_2_S	ppm
Hydraulic Retention Time	days
Biogas Production	L
FOS/TAC	-

**Table 6:** Statistical overview of the dataset employed in this study.

Parameter	Mean	Std Dev	Min	25%	50%	75%	Max
Equalisation Tank pH	6.18	0.57	4.99	5.80	6.25	6.64	7.47
Equalisation Tank COD	15,939	4299	4900	13,996	15,462	17,914	32,948
Feed Volume	303	106	0	240	288	400	500
Organic Loading Rate	5.28	2.08	0	3.96	5.00	6.58	13.37
Reactor pH	7.22	0.21	6.50	7.10	7.21	7.31	7.70
Reactor COD	4158	1725	1761	2644	3682	5117	7834
Current	2162	716	240	1866	2283	2515	3990
Temperature	32.79	3.10	20.3	31.19	33.97	34.80	36.80
CH_4_ Percentage	68.09	4.29	51.6	65.7	67.8	70.4	82.0
H_2_S	687	383	7	427	632	952	1942
Hydraulic Retention Time	3.30	1.83	0	2.30	3.10	3.80	16.1
Biogas Production	1569	625	254	1099	1387	2078	3128
FOS/TAC	0.43	0.22	0.10	0.29	0.38	0.53	1.29

## Data Availability

All data underlying the findings of this review are unrestricted and fully available. Data supporting the findings of this study can be obtained through contact with the author.

## Abbreviations

The following abbreviations are used in this manuscript:

AD	Anaerobic digestion
ANN	Artificial neural network
CNN	Convolutional neural network
COD	Chemical oxygen demand
DNN	Deep neural network
DT	Decision tree
MEC-AD	Microbial electrochemical cell anaerobic digestion
ML	Machine learning
MLP	Multilayer perceptron
RF	Random forest
SVM	Support vector machine
SVR	Support vector regression
TS	Total solids
TSS	Total suspended solids
VFA	Volatile fatty acids
XAI	Explainable AI
